# Non-invasive identification of mesenchymal glioblastoma using quantitative radiomic features from advanced diffusion MRI: a preclinical-to-clinical transfer learning strategy

**DOI:** 10.1186/s41747-025-00652-4

**Published:** 2025-11-14

**Authors:** Alberto L. Gallotti, Nicolò Pecco, Valentina Pieri, Manuela Cominelli, Gianluca Brugnara, Luisa Altabella, Ilaria Pagano, Marcella Callea, Andrei Fodor, Filippo Gagliardi, Pietro Mortini, Pietro L. Poliani, Andrea Falini, Antonella Castellano, Rossella Galli

**Affiliations:** 1 Neural Stem Cell Biology Unit, Division of Neuroscience, IRCCS San Raffaele Scientific Institute, Milan, Italy; 2 Neurosurgery and Gamma Knife Radiosurgery Department, IRCCS San Raffaele Scientific Institute, Milan, Italy; 3 Vita-Salute San Raffaele University, Milan, Italy; 4 Neuroradiology Unit and CERMAC, IRCCS San Raffaele Scientific Institute, Milan, Italy; 5 Pathology Unit, Molecular and Translational Medicine Department, University of Brescia, Brescia, Italy; 6 Pathology Unit, IRCCS San Raffaele Scientific Institute, Milan, Italy; 7 Radiotherapy Unit, IRCCS San Raffaele Scientific Institute, Milan, Italy

**Keywords:** Diffusion magnetic resonance imaging, Glioblastoma, Heterografts, Molecular classification, Radiomics

## Abstract

**Background:**

Glioblastoma (GBM) is no longer regarded as a single disease, as distinct molecular subgroups exist, with the mesenchymal (MES) having the worst prognosis. As such, there is a critical need for noninvasive methods to determine GBM molecular status. Although conventional magnetic resonance imaging (MRI)-based radiomics showed promise for predicting GBM characteristics, few studies evaluated pipelines that leverage advanced diffusion MRI (dMRI) techniques, such as diffusion tensor imaging (DTI) and neurite orientation dispersion and density imaging (NODDI), enabling characterization and quantification of tumor microstructure.

**Materials and methods:**

To identify advanced dMRI radiomic features specific to MES GBM, we enrolled 36 GBM patients (4 mesenchymal, 32 non-mesenchymal), who underwent presurgical DTI and NODDI protocols. Post-surgery samples were processed to establish subgroup-specific GBM sphere-forming cell (GSC) lines, generating 21 xenografts (12 non-mesenchymal, 9 mesenchymal) that were subjected to the same dMRI protocols.

**Results:**

By leveraging a preclinical-to-clinical transfer learning approach, a machine learning classification algorithm was developed to generalize between preclinical and clinical contexts. Models were trained on xenograft-derived data and validated using an independent patient test set. Using bootstrap resampling to estimate confidence intervals, the XGBoost model achieved an area under the receiver operating characteristic curve of 0.93 (95% confidence interval (CI): 0.79–1.00) and a balanced accuracy of 0.86 (0.64–1.00) for MES prediction. A subset of 9 selected features was sufficient to build a model that accurately predicted MES affiliation.

**Conclusion:**

DTI and NODDI radiomics revealed key features that predict MES GBM and correlate with biological and clinical characteristics.

**Relevance statement:**

A DTI and NODDI-based model trained on preclinical xenograft-derived data can be validated in a human patient cohort, demonstrating cross-species generalizability of radiomic biomarkers. This approach provides a noninvasive means to molecularly stratify GBM patients, enabling the potential to inform tailored treatment.

**Key Points:**

- We defined a machine learning algorithm that, starting from subgroup-specific glioblastoma xenografts, reliably identifies the mesenchymal affiliation of glioblastoma patients.
- The specific dMRI features selected from experimental preclinical models of glioblastoma hold a remarkable predictive value.
- The same features provide insights into subgroup-restricted tumor tissue microstructure and its relationship with the malignant behavior of mesenchymal glioblastomas.

**Graphical Abstract:**

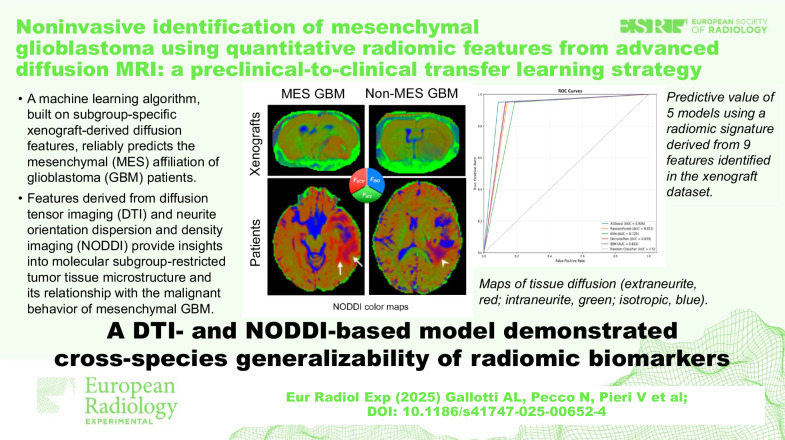

## Background

Glioblastoma (GBM) is the most common and malignant primary brain tumor of adults [1, 2], with a very severe prognosis of 15–18 months [3, 4]. Molecular features have been incorporated into the 2021 World Health Organization classification of brain tumors, based on which GBMs are diagnosed as such when presenting as a diffuse astrocytic tumor with isocitrate dehydrogenase (IDH) wild-type status, telomerase reverse transcriptase promoter mutation, epidermal growth factor receptor (EGFR) amplification, or +7/-10 chromosomal aberrations [5–8].

In the last decades, distinct bulk transcriptional analyses identified three main, ‘conventional’ GBM subtypes, namely proneural (PN), classical (CL) and mesenchymal (MES), which are endowed with specific gene signatures and different clinical features [9, 10]. The most malignant MES GBMs are identified by the expression of chitinase-3-like protein 1 (YKL40), MET proto-oncogene (MET), and cluster of differentiation 44 (CD44), by neurofibromin 1 deletions, by activation of NFkB, and by the presence of a significantly increased immune cell infiltrate [9–13]. MES GBM patients are more resistant to alkylating and antiangiogenic agents as well as to radiotherapy, and show, therefore, a shorter overall survival [9, 13]. MES GBMs are also associated with the activation of lipidic and aminoacidic pathways that contribute to the so-called glycolytic/plurimetabolic phenotype that results in resistance to multiple treatment strategies [14]. Moreover, transition among different transcriptional states is often observed upon recurrence, with the acquisition of either glycolytic/plurimetabolic /mesenchymal [14] or neuronal-like phenotypes [15].

Given the differences in malignancy and treatment resistance of the subgroups [9, 12], the identification of robust subgroup-specific surrogate markers may be helpful to predict prognosis, develop personalized therapies and monitor treatment follow-up. As an example, the *bona fide* identification of MES affiliation before surgery might translate into different radicality intent of the neurosurgeons, as gross total resection was found to be advantageous for non-MES GBMs but not for MES ones [16].

To date, the costs of genetic profiling and the scarcity of the tumor tissue obtained from biopsies of patients not eligible for surgery and required for immunohistochemistry analysis are important constraints for an effective translation of molecular features into both diagnostic and follow-up settings. An alternative is the implementation of methodologies to noninvasively define the tumor molecular status. Magnetic resonance imaging (MRI) is the technique of choice to diagnose and stage GBMs. The integration of imaging-derived features with molecular data has given rise to radiogenomics, to correlate radiomic features with gene expression profiles and/or to specific mutations [17–19].

Most radiogenomics studies integrated molecular information with features extracted from conventional MRI [20–23]. However, advanced MRI protocols may provide more accurate information on tumor tissue microstructure than conventional techniques [24–26]. Among the former diffusion MRI releases detailed information about tissue microstructure after appropriate signal elaboration through either parametric methods (*e.g*., diffusion tensor imaging, DTI) or mathematical models (*e.g*., neurite orientation dispersion and density imaging, NODDI). By sampling the diffusion signal in multiple directions, DTI generates a tensor, *i.e*., a multidimensional vector determining quantitative parameters, such as mean diffusivity (MD) and diffusion fractional anisotropy (FA) [27].

Although DTI has proven useful in identifying risk groups with dysregulated biological pathways in gliomas [28], it suffers from limitations recently overcome by the NODDI model, which enables a more detailed tissue characterization than the classical DTI metrics. NODDI quantitatively estimates the relative contribution of three distinct diffusion compartments to the total diffusion signal in each voxel [29]: the isotropic fraction (F_ISO_), anisotropic Gaussian (extracellular volume fraction, F_ECV_), and anisotropic non-Gaussian (intracellular volume fraction, F_ICV_) diffusion, which approximate cerebrospinal fluid-like, extraneurite and intraneurite compartments, respectively [30]. When NODDI is applied to GBM, the F_ISO_ component approximates vasogenic edema and colliquated necrosis, F_ECV_ describes the tumor infiltrative edema, and F_ICV_ depicts cellular edema due to acute ischemia or extremely compacted cellularity [31].

We aimed at identifying radiomic features from DTI- and NODDI-derived maps that may discriminate MES affiliation in molecularly stratified GBM patients starting from xenografts generated by orthotopically implanting GBM sphere-forming cells (GSCs) isolated from the same patients’ tumor. These *in vivo* models, although faithfully resembling their tumor of origin, are more molecularly and histologically homogeneous than their clinical counterpart [32–36]. To generalize the preclinical imaging biomarkers to clinical settings, a transfer learning framework was implemented by using radiomic features extracted from GBM xenograft models used to train machine learning models, which were then validated on an external human patient cohort.

## Materials and methods

### Generation of glioblastoma sphere-forming cell (GSC) lines

To establish GSC lines, a portion of each post-surgery GBM specimen was cultured under the NeuroSphere Assay conditions [37], as also detailed in the Supplementary Methods section.

### Generation of GSC-derived xenografts

NOD.Cg-Prkdc^scid^Il2rg^tm1Wjl^/SzJ (NOD SCID GAMMA, NSG) mice (Charles River Laboratories; RRID: IMSR_JAX:005557) were used as xenograft recipients. Four × 10^5^ subgroup-specific GSCs were transferred in 4 µL of Dulbecco’s Modified Eagle Medium (DMEM) containing Deoxyribonuclease (DNase) and delivered into the right striatum of deeply anesthetized, NSG mice at 6–8 weeks of age by stereotactic injection through a 5-µL Hamilton micro-syringe at a flow rate of 0.5 µL/min. The following coordinates were used: antero-posterior: 0; medio-lateral: 2.5 mm; dorso-ventral: 3 mm. Animals were sacrificed at different time points after transplantation, as monitored by longitudinal MRI.

#### Transcriptional subgroup affiliation by immunohistochemistry

Staining for subgroup-specific gene classifiers, such as Achaete-Scute Family BHLH Transcription Factor 1 (ASCL1), Oligodendrocyte Transcription Factor 2, PDGFRα, p53 for PN GBM, EGFR for CL GBM, and MET, pNDRG1 and Chitinase-3-like protein 1 (*YKL40*) for MES GBM, was performed as described [38] and thoroughly detailed in the Supplementary Methods section.

#### Patient and xenograft MRI acquisition

GBM patient datasets were acquired on a 3-T Ingenia CX scanner (Philips Healthcare), using a 32-channel head coil. Xenograft MRI acquisition was carried out on a 7-T preclinical scanner (Bruker, BioSpec 30/70 USR, Paravision 5.1) at the Ospedale San Raffaele Experimental Imaging Centre, equipped with 450/675 mT/m gradients. Acquisition protocols and image preprocessing are detailed in the Supplementary Methods section. For image acquisition parameters, refer to Supplementary Material.

#### MRI image preprocessing

All NODDI volumes were corrected for movement and eddy-current distortions, using the “eddy” tool of Functional Magnetic Resonance Imaging of the Brain (FMRIB) (Oxford Centre) Software Library (FSL, University of Oxford, https://fsl.fmrib.ox.ac.uk/fsl/ RRID:SCR_002823). Once preprocessing was completed, the Watson-NODDI model was fitted to the two-shell diffusion MRI datasets using the MATLAB (RRID:SCR_001622) NODDI toolbox (http://mig.cs.ucl.ac.uk/Tutorial.NODDImatlab; RRID:SCR_006826) to extract the F_ECV_, F_ICV_, F_ISO_ and orientation dispersion index (ODI) maps [39]. The output compartments were reparameterized so that the sum of F_ECV_, F_ICV_, and F_ISO_ equaled 1 in each voxel [31, 39]. NODDI compartment maps were also combined into a single four-dimensional red-green-blue image (red for F_ECV_, green for F_ICV_, and blue for F_ISO_) for visualization and quality-check purposes.

FSL (RRID:SCR_002823) built-in “dtifit” tool was separately applied to DTI shell (DTI: 35 directions at *b*-value 711 s/mm^2^) to estimate the diffusion tensor, and to generate FA and MD tensorial maps [39]. FSL Brain Extraction Tool (bet) was used to skull-strip the three-dimensional fluid-attenuated inversion-recovery (FLAIR) image as well as the DTI *b* = 0 image. Manual segmentation of tumor lesions was performed on the FLAIR images in patients and on the DTI *b* = 0 images in xenografts using ITKSNAP software [40]. Skull-stripped three-dimensional FLAIR and relative derived-FLAIR tumoral region of interest (ROI) were co-registered to the DTI *b* = 0 image through an affine transformation [39].

#### Radiomic feature extraction

To improve reproducibility and standardize texture analysis in all directions, patient NODDI and DTI scans, with an average in-plane voxel resolution of 1.95 × 1.95 mm^2^ and an average slice thickness of 2.16 mm, were resampled to 2 × 2 × 2 mm^3^ to obtain isotropic voxels. Similarly, xenograft DTI scans, with an in-plane voxel resolution of 0.11 × 0.11 mm^2^ and a slice thickness of 0.75 mm, were resampled to isotropic voxels of dimension 0.11 mm^3^. All tumoral ROI underwent a gray-level intensity filtering step, where voxels with intensities outside the range [mean ROI -3 standard deviation, mean ROI + 3 SD] were excluded from texture analysis. This procedure minimizes the impact of outliers and acquisition-related intensity variations [41]. Quantization of gray levels was performed by decreasing the number of gray levels to 6 bits/pixel, meaning 64 levels of gray.

Radiomic feature extraction was performed using Pyradiomics (v2.2.0, http://www.radiomics.io/pyradiomics.htm). For each diffusion-derived map (DTI and NODDI) by using the three-dimensional FLAIR masks in patients and the *b* = 0 image in xenografts, we extracted a comprehensive set of features encompassing first-order intensity statistics and texture features, including gray-level co-occurrence matrix (GLCM), gray-level run-length matrix (GLRLM), gray-level size-zone matrix (GLSZM), neighboring gray tone difference matrix (NGTDM), and gray-level dependence matrix (GLDM). The counts and detailed descriptions of the extracted features, grouped by category, are provided in Supplementary Methods: “Radiomics features paragraph.”

#### Radiomic feature selection

After radiomic feature extraction in both patients and xenografts, feature dimensionality reduction was performed exclusively on the xenograft dataset through a two-step procedure. First, multicollinearity was addressed by iteratively removing features with a variance inflation factor (VIF) greater than 10, using R software (RRID:SCR_001905). At each iteration, VIF values were recalculated, and the feature with the highest VIF was removed until all remaining features had VIF ≤ 10. Second, the output set of features from the VIF step was subjected to univariate feature selection using the F_classif analysis of variance (ANOVA) test implemented in Scikit-Learn (Python, RRID:SCR_008394). Features with *p*-values < 0.05 were retained as the final feature set for model development, ensuring both low redundancy and discriminatory power for the two GBM subgroups (MES *versus* non-MES).

### Model development

Different machine learning approaches, such as k-nearest-neighbor, support vector machines, random forest, eXtreme Gradient Boosting (XGBoost) and decision tree, were tested. Each classifier was trained on the xenograft dataset with 4-fold cross-validation and an inner loop for hyperparameter tuning. The same normalized subset of features identified on the xenograft dataset was pooled and used for testing on the patient hold-out dataset (Supplementary Methods: “Model development”).

Given the limited size of the training dataset, a randomized label control experiment was performed to assess the robustness of the predictive model and to confirm that it identified meaningful patterns within the data rather than relying on spurious correlations. Before training, the labels of the training dataset were randomly shuffled, effectively removing any inherent relationship between the input features and the target labels. The same shuffled model was then trained on this randomized dataset and evaluated on the patients’ hold-out set. This approach serves as a sanity check to verify that the model performance is not driven by chance or artifacts within the dataset. Finally, the SHapley Additive exPlanations (SHAP) values [42] were used to quantify the contribution of individual features when predicting patients’ hold-out test set. This approach uses SHAP values as a model-agnostic, objective metric to quantify the contribution of each feature to the model’s predictions, ensuring consistency and interpretability across models.

### Statistical analysis

Association between overall survival (OS) and GBM molecular classification was analyzed by Kaplan–Meier and Log-rank. Feature reduction was assessed by using the variance inflation factor (VIF) using a threshold of 10. Model performances were evaluated on the patient test dataset by using bootstrap resampling (1,000 iterations) to estimate 95% confidence intervals (CIs) for balanced accuracy, precision, recall, area under the receiver operating characteristic curve (AUROC), weighted Fbeta-score and the Matthew’s correlation coefficient. To ensure meaningful estimates given the small MES subgroup, we filtered bootstrap samples to include only those with at least two MES cases. Statistical analyses were run with the ScikitLearn Fclassif algorithm that performs a test for group differences. A two-tailed *p*-value < 0.05 was considered statistically significant.

## Results

### GBM molecular classification

According to the inclusion and exclusion criteria, we enrolled 36 IDH 1/2 wild-type GBM patients (Supplementary Schematic S1 and Supplementary Results). Transcriptional subgroup affiliation was determined by means of an immunohistochemistry-based algorithm that predicts the GBM transcriptional subtypes with high accuracy [38]. Expression of subgroup-specific gene classifiers (Supplementary Fig. S1) was scored in a semiquantitative manner on multiple areas of the same GBM post-surgery specimen based on the percentage of immunoreactive neoplastic cells.

Based on this analysis, 36 GBM patients were classified as 4 MES (11.1%) and 32 non-MES (88.9%), the latter including 13 CL (36.1%), 11 PN (30.6%), 6 PN/CL (16.6%), and 2 mixed PN/CL/MES GBM (5.6%) (Supplementary Schematic S1 and Supplementary Table S1). Given the higher malignancy and worse clinical behavior of MES GBMs, we focused our radiomics analysis on predicting such a subtype against all the others, which we collectively grouped under the definition ‘non-MES.’

The mean overall survival for all GBM patients was 13.64 ± 11.68 months. Kaplan–Meier curves for GBM patients subdivided into MES *versus* non-MES GBM (Supplementary Fig. S2) indicated that MES GBM patients had a significantly worse prognosis when compared to the other subgroups.

### Generation of xenografts through orthotopic transplantation of GSCs isolated from the same patients’ surgical specimens

To isolate and long-term expand *in vitro* patient-specific GSC lines, we subjected GBM specimens to the selective culture conditions of the Neurosphere Assay. GSC lines were established and validated *in vitro* as being self-renewing and multipotent [43]. Notably, extensive culturing of GSCs under the NeuroSphere Assay conditions is known to enrich for cells affiliated to a single, predominant molecular subgroup, as determined by bulk transcriptomics [12, 44–48]. When we transplanted subgroup-restricted GSCs orthotopically into NSG mice, they gave rise to xenografts (*n* = 21) that, after being subjected to the same histological analysis as patients’ specimens (Supplementary Schematic S1 and Supplementary Fig. S3), were classified as follows: 9/21 (42.9%) were MES and 12/21 (57.1%) non-MES, the latter including 8 CL (38.1%), 3 PN (14.3%) and 1 PN/CL (4.7%) GBMs. Thus, similar to their corresponding GSC lines, most xenografts were characterized by the affiliation to a unique and prevalent molecular subgroup [45].

### MRI analysis and radiomic feature extraction

**Fig. 1:**
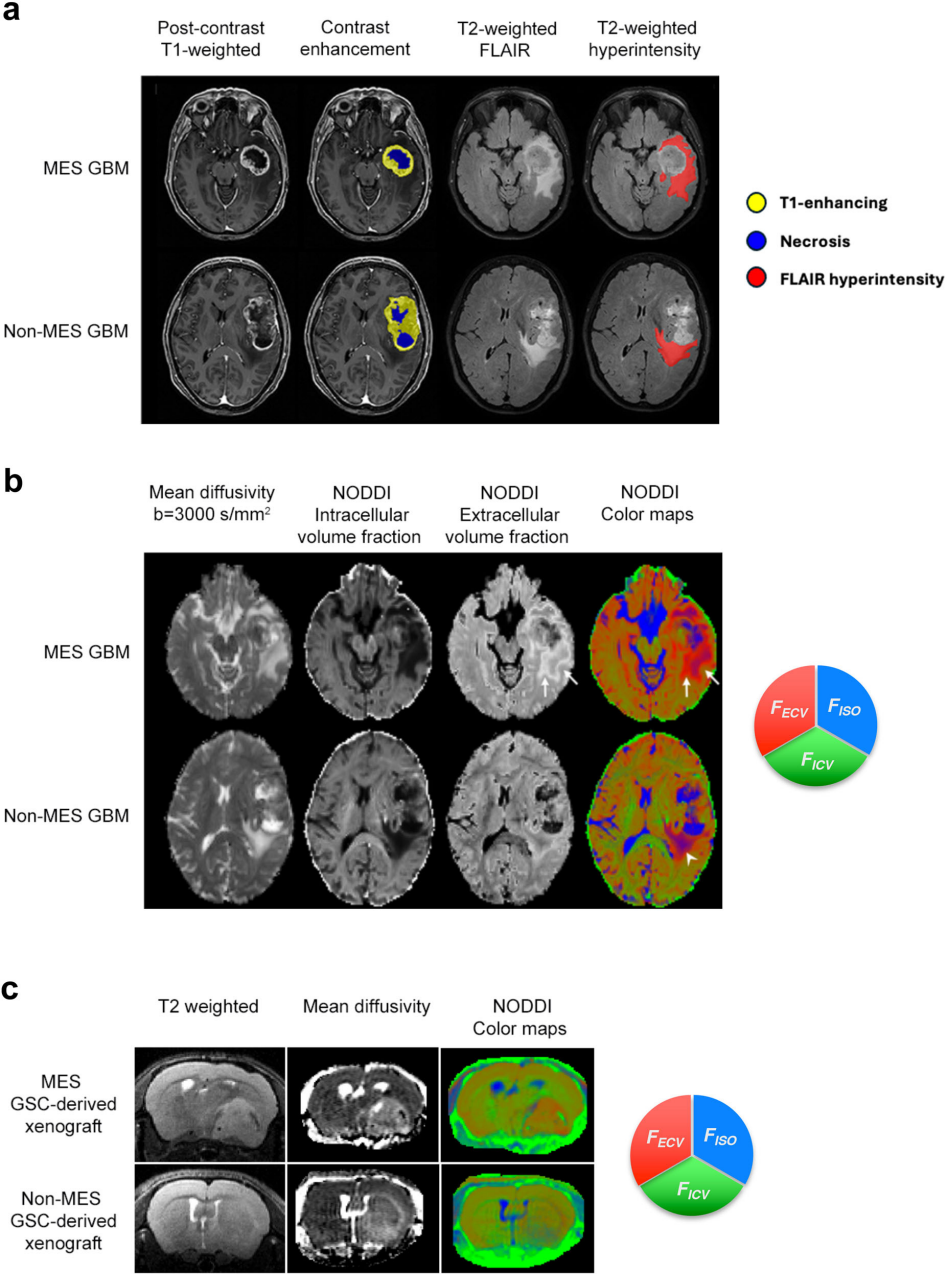
Advanced diffusion MRI identifies subgroup-specific features in glioblastoma patients and GSC-derived xenografts. **a** Conventional MRI images. Yellow: contrast enhancement; blue: central necrosis; red: FLAIR hyperintense regions of interest. **b** Advanced MRI NODDI maps show the different components of tissue diffusion, and the NODDI color map summarizes the relative contributions of the different microstructural compartments: red for extracellular volume fraction (F_ECV_), green for intracellular (or intraneurite) volume (F_ICV_) fraction, and blue for isotropic Gaussian volume (F_ISO_) fraction. Note the increase of F_ECV_ in the MES tumor (white arrows) and the increase of the F_ISO_ in the non-MES tumor (white arrowheads). **c** Typical growth pattern of MES and non-MES GSC-derived xenografts. MES xenografts are visible in T2-weighted images, mean diffusivity sequences, and NODDI color maps as large and compact masses. Conversely, non-MES xenografts are very infiltrating and visible only in MD and NODDI color maps. GSC, Glioblastoma sphere-forming cell; MES, Mesenchymal; MRI, Magnetic resonance imaging; NODDI, Neurite orientation dispersion and density imaging; Non-MES, Non-mesenchymal

All patients were subjected to conventional (Fig. 1a) and advanced (DTI and NODDI) (Fig. 1b) preoperative MRI (Supplementary Schematic S1). Quantitative NODDI parameters, such as F_ICV_, F_ECV_ and F_ISO_, highlighted a distinctive imaging pattern for the two subtypes (Fig. 1b), with MES tumors showing an increase in F_ECV_.

Likewise, mice bearing subgroup-specific xenografts were subjected to the same sequences used for patients (Supplementary Schematic S1). Whereas non-MES xenografts were highly infiltrating and, therefore, detectable only by mean diffusion sequences and not by T2- or post-contrast T1-weighted sequences (Fig. 1c), MES GSC-induced xenografts grew as expanding masses that were detectable by all sequences [45–47] (Fig. 1c). Most remarkably, when subjected to NODDI analyses, subgroup-specific xenografts showed distinctive imaging patterns, with MES xenografts showing a significantly higher F_ECV_ than non-MES xenografts (Fig. 1c).

For radiomic feature extraction, since contrast-enhancing ROIs were not available for non-MES xenografts, ROIs from human 3D-FLAIR and from xenograft *b* = 0 masks were used for DTI and NODDI maps (Supplementary Schematic S1). A total of 91 features was extracted for each map, leading to 546 features for three-dimensional FLAIR ROI masks in patients and 546 features for ROI masks in xenografts.

#### Radiomic feature selection

**Fig. 2:**
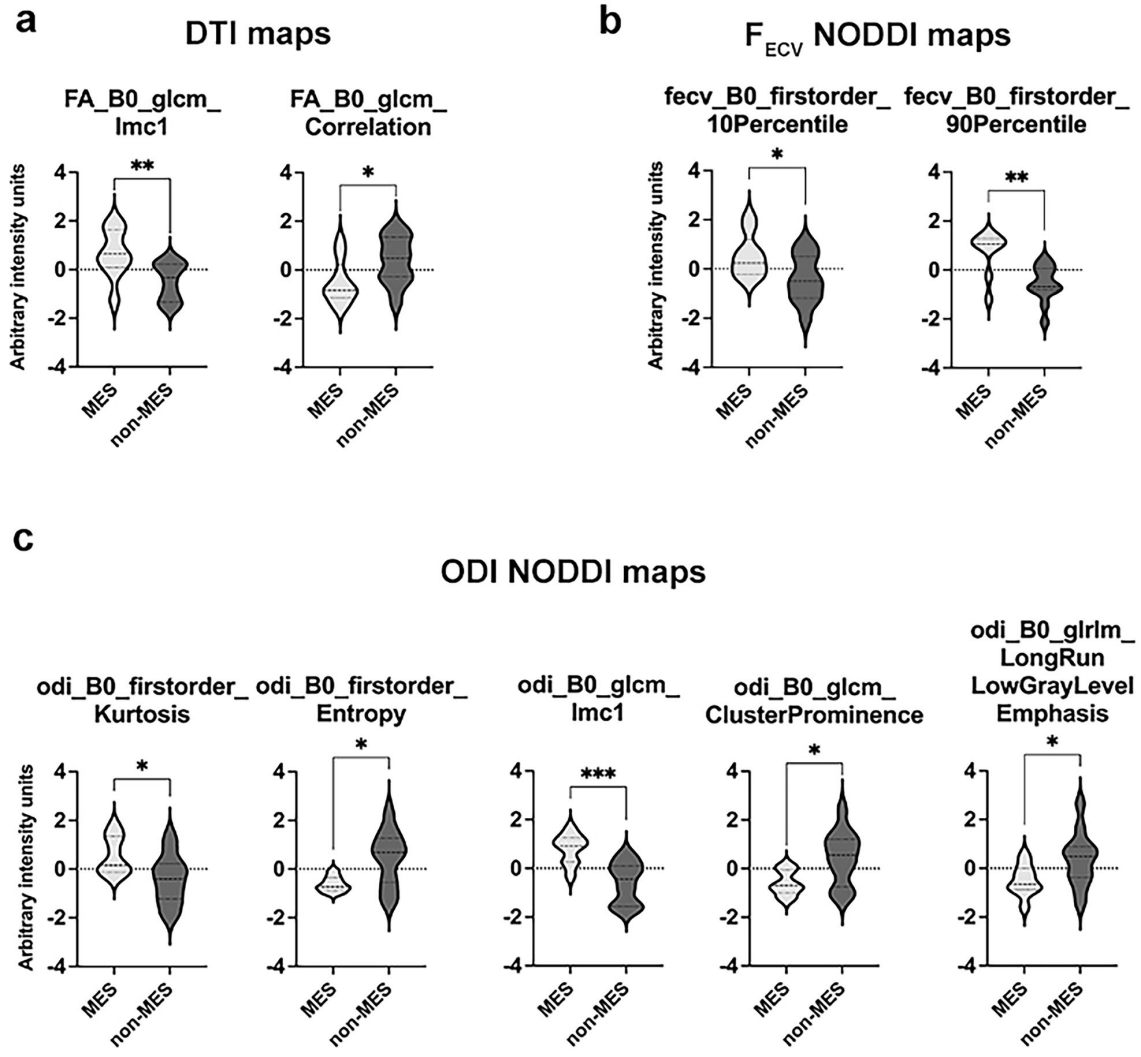
Radiomic features selection in mesenchymal and non-mesenchymal glioblastomas. **a** Significant diffusion tensor imaging radiomic features discriminating MES from non-MES GSC-derived xenografts. **b**, **c** Significant F_ECV_ and ODI NODDI radiomic features discriminating MES from non-MES GSC-derived xenografts. Quantitative data are represented as violin plots, with interquartile ranges and median line. Student’s *t*-test, unpaired, two-tailed. * *p* < 0.05; ** *p* < 0.01; *** *p* < 0.005; **** *p* < 0.001. FA, Fractional anisotropy; F_ECV_, Fraction of extraneurite volume; GLCM, Gray-level co-occurrence matrix; GLSZM, Gray-level size zone matrix; GLRLM, Gray-level run-length matrix; GSC, Glioblastoma sphere-forming cell; IMC1, Informational measure of correlation 1; ODI, Orientation dispersion index; MES, Mesenchymal; NODDI, Neurite orientation dispersion and density imaging; Non-MES, Non-mesenchymal

After radiomics feature extraction, multicollinearity was reduced using the VIF algorithm, resulting in the exclusion of 489 redundant features. The remaining features were then subjected to univariate feature selection to identify those most discriminative between MES and non-MES GBM subgroups in the xenograft dataset, yielding 9 significant features (Supplementary Schematic S1 and Supplementary Table S2). Informational measure of correlation 1 (IMC1) and correlation were both derived from DTI-FA maps and calculated on the gray-level co-occurrence matrix (GLCM), which defines the second-order joint probability function of a region of interest (ROI) by considering the spatial relationship between voxels (Fig. 2a). The remaining features were all extracted from NODDI maps (Fig. 2b, c). F_ECV_-derived NODDI features described the 10th and 90th percentile that collect, respectively, the lower and upper decile gray values and were identified as significant in the NODDI extracellular hindered diffusion (Fig. 2b). The other five features were derived from the orientation dispersion index (ODI) map and showed divergent trends between MES and non-MES GBM xenografts (Fig. 2c). For a description of the selected features, please refer to Supplementary Material: “Interpretation of selected radiomic features.”

### Diagnostic performance of the radiomic-based prediction models

The identified nine common features were then used to train five distinct prediction models, namely support vector machine, k-nearest neighbor, random forest, eXtreme Gradient Boosting (XGBoost) and decision tree (Supplementary Schematic S1). We used the xenograft dataset (12 non-MES *versus* 9 MES GBM xenografts) as training set, due to the highly divergent phenotypes of subgroup-restricted xenografts that are suitable for identifying consistent thresholds for the models. Then, we exploited the patient dataset as the hold-out test (32 non-MES *versus* 4 MES GBMs).

**Fig. 3:**
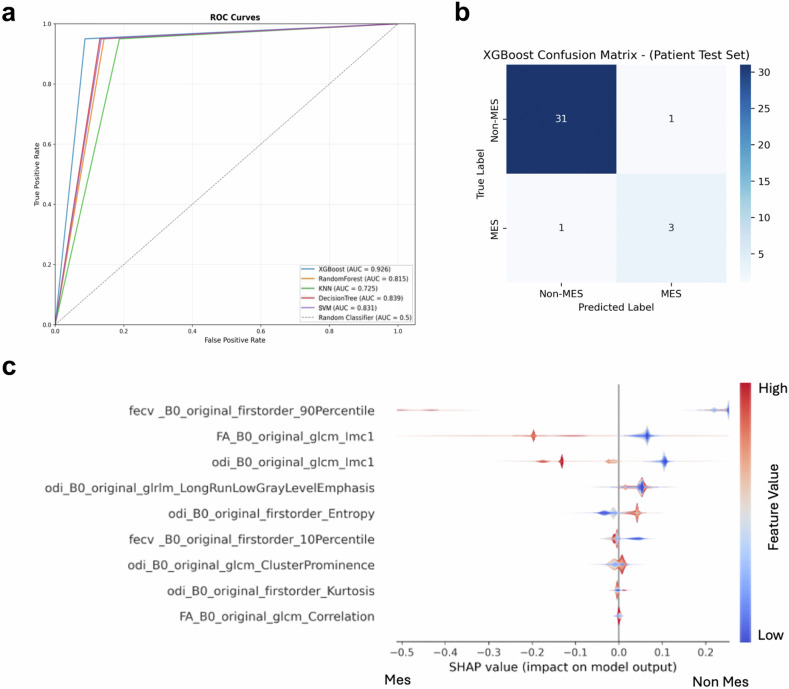
GBM transcriptional subgroups can be predicted by an advanced diffusion radiomic signature. **a** Receiver operator characteristic curves showing the predictive power of the radiomic signature by five different prediction models based on the nine radiomic features identified in the xenograft dataset and tested on the patient dataset. **b** Confusion matrix for the best-performing XGBoost model on the hold-out patient test set, illustrating sensitivity for MES identification and misclassification rates. **c** The contribution of individual features for the XGBoost model when predicting the patient’s test set is shown as layered violin plots using SHAP values. KNN, k-nearest neighbor; MES, Mesenchymal; Non-MES, Non-mesenchymal; SVM, Support vector machine; XGBoost, eXtreme gradient boosting

Overall, the performance of all models was good, with the XGBoost model showing the best performance metrics (Table 1). Accordingly, the XGBoost model achieved an AUROC of 0.926 (95% CI: 0.793–1.000), a balanced accuracy of 0.860 (95% CI: 0.636–1.000), and an F1-macro score of 0.851 (95% CI: 0.636–1.000), with confidence intervals estimated via 1,000-iteration bootstrap resampling on the patient test dataset (Fig. 3a, b). Furthermore, the inclusion of NODDI-derived features substantially improved discriminative performance, highlighting their complementary value to DTI features (see Supplementary Table S3).

**Table 1:** Performance of the five prediction models in predicting the molecular subgroup glioblastomas based on common selected magnetic resonance imaging features on patient’s dataset

	XGBoost	Random forest	KNN	Decision tree	SVM
Balanced accuracy	0.860 (0.636–1.0)	0.704 (0.440–0.970)	0.632 (0.500–0.875)	0.781 (0.545–0.955)	0.799 (0.539–0.956)
Precision macro	0.871 (0.636–1.0)	0.673 (0.453–0.955)	0.802 (0.417–0.986)	0.655 (0.521–0.818)	0.680 (0.520–0.859)
Recall macro	0.860 (0.636–1.0)	0.704 (0.440–0.970)	0.632 (0.500–0.875)	0.781 (0.545–0.955)	0.799 (0.539–0.956)
F1 macro	0.851 (0.636–1.0)	0.673 (0.446–0.893)	0.660 (0.455–0.921)	0.669 (0.485–0.859)	0.699 (0.500–0.883)
MCC	0.720 (0.273–1.0)	0.367 (-0.097 to 0.804)	0.400 (0.000–0.853)	0.412 (0.058–0.750)	0.456 (0.057–0.767)
AUROC	0.926 (0.793–1.0)	0.815 (0.617–0.992)	0.725 (0.491–0.945)	0.839 (0.672–0.955)	0.831 (0.616–0.990)

Data are given as mean (95% confidence interval). Models were tuned on xenograft values (train dataset) and tested on patients (test dataset)

*AUROC* Area under the receiver operating characteristic curve, *KNN* k-nearest neighbor, *MCC* Matthews’ correlation coefficient, *SVM* Support vector machine, *XGBoost* eXtreme gradient boosting

When evaluating the model with a randomized label control test, a substantial drop in performance as compared to the model trained with the correct labels was observed, indicating that the original model’s predictive capability was likely derived from genuine patterns within the data (Supplementary Table S4 and Supplementary Fig. S4).

#### Best model explanation

The contribution of individual features in predicting the subgroup affiliation in the patient’s hold-out test set was quantified using SHAP values. The layered violin plots illustrate the direction and the strength of the impact of each feature on the model’s output when predicting the patient’s dataset using the XGBoost model (Fig. 3c and Supplementary Table S5). The top features that had the greatest impact in the patient test set were three, namely the 90th percentile from the NODDI-F_ECV_ map and the IMC1 derived from the GLCM matrix on the FA and ODI map, whose higher values were associated with the MES subgroup.

## Discussion

Since MES GBM patients exhibit the shortest survival and increased treatment resistance [9, 12, 13], a noninvasive determination of specific radiomic features might be relevant to stratify prognosis and inform treatments.

Many studies developed radiomic pipelines based on conventional MRI acquisitions of GBM to predict IDH and 1p/19q codeletion status [49], profile the immune cell infiltrate [50], determine proliferation index and tumor grade, and differentiate recurrence from radionecrosis [19, 51–53]. Some groups performed radiomic analyses on T2-weighted FLAIR and contrast-enhanced T1-weighted sequences, extracting features from enhancing nodule, nonenhancing tumor core, and peripheral edema, managing to accurately predict transcriptional affiliation [22, 54].

Conversely, little is known about the relevance of quantitative features extracted from advanced protocols in defining subgroup-specific GBM microarchitecture based on water molecule diffusion in a non-Gaussian probability distribution. In fact, although Gaussian diffusion analysis, such as DTI, has been successfully employed for the deep learning-based extraction of features able to enhance glioma stratification and identify risk groups [28], innovative non-Gaussian diffusion models that may approximate brain structures better than DTI, such as NODDI, have not been implemented yet. The few studies in the literature to adopt both DTI and NODDI to characterize brain tumors did not apply radiomic pipelines and focused on different tasks, such as discriminating GBMs from metastases, reporting higher NODDI F_ECV_ component in the perinodular area of GBMs and higher F_ISO_ around metastases, with NODDI being more sensitive than apparent diffusion coefficient and FA [55, 56]. Likewise, other studies reported that IDHwt gliomas had significantly lower minimum MD values and maximal FA and NODDI F_ICV_ as compared to IDH-mutant counterparts, with a significant correlation between DTI metrics and IDH status [57]. Notably, higher ODI values suggest greater dispersion of fibers in IDH wild-type gliomas, whereas increased extracellular water in IDH-mutant lesions may reduce FA signal [57]. Concurrently, no difference was reported in NODDI metrics pertaining to IDH status. However, gliomas with high F_ICV_ in the tumor parenchyma and low in its periphery were more likely to be higher-grade, while lower-grades showed an opposite trend [58].

To assess this gap, we recruited 36 patients whose GBMs were affiliated with each of the three molecular subgroups or with a combination of them. To identify radiomic features that may have predictive power in GBM patients, we took advantage of xenografts generated through the orthotopic transplantation of subgroup-specific GSC lines established from the same patients and subjected to similar advanced MRI protocols.

Significant features able to discriminate between MES from non-MES GBM were identified in xenografts. We identified nine features in the xenograft dataset, two of them being determined by DTI and the remaining by NODDI. Notably, among the three most significant features in the SHAP analysis, one was derived from DTI-FA and two from NODDI. Specifically, the DTI-FA IMC1 feature was calculated on the GLCM matrix, which defines the second-order joint probability function of a ROI by considering the spatial relationship of voxels. Basically, GLCM features describe an image texture by calculating how often pairs of voxels with specific values and in a specific spatial relationship occur [53, 59–62]. The DTI-FA IMC1 feature was significantly higher in MES than in non-MES tumors, suggesting MES GBMs as more heterogeneous in texture.

The 90th percentile values in the NODDI-F_ECV_ map were also quantitatively higher in MES than in non-MES xenografts, indicating a hindered diffusion component. Since, under pathological conditions, the F_ECV_ component provides an approximation of infiltrative edema [31], MES xenografts may seem more highly invasive locally than non-MES tumors. Remarkably, however, MES xenografts appear endowed with better-defined boundaries, while non-MES lesions tend to disperse along white matter tracts [45–47]. This might appear in contrast with our observations. However, these features only describe the tumor nodule and its immediate surroundings, where alterations are strong enough to generate *b* = 0 anomalies, with no indications on what happens farther away. Thus, higher local infiltration of MES tumors may be explained by an overrepresentation of fast proliferating, slow migrating, and less invasive cells, while non-MES cells could show an opposite and more motile behavior that counteracts accumulation in nodule surroundings [63–65]. Alternatively, overproduction of extracellular matrix by MES GBMs may alter anisotropy, as reported when aiming to differentiate GBMs from metastases [56]. Other reports showed that F_ECV_ values are higher in tumor ROIs with respect to normal brain, due to vasogenic and infiltrative edema [30, 31].

The ODI IMC1 has a mining similar to the corresponding DTI-FA map, suggesting a more spatially coherent pattern of orientation dispersion. This may reflect consistent microstructural disruption across the tumor volume, possibly linked to higher cellular density and extracellular matrix remodeling. Of note, the IMC1 feature appears to be higher in MES tumors on both FA and ODI maps. While FA and ODI often reflect opposing aspects in a healthy tissue context, in pathological contexts like tumors, they can exhibit high texture complexity, leading to similar behavior in radiomic features like IMC1, thereby capturing heterogeneity and not simply intensity direction. Thus, MES GBMs may be endowed with a more textural complexity in both diffusion anisotropy and neurite dispersion patterns and with greater microstructural heterogeneity, consistent with their aggressive and infiltrative phenotype. This convergence across FA and ODI modalities strengthens the pathological relevance of our findings, implying that IMC1 may be capturing a common hallmark of MES tumors, that is, their spatially disorganized and heterogeneous tissue architecture.

To develop an algorithm able to generalize between preclinical and clinical settings, we trained five different models by training the model on xenografts and testing the performance on the patient test set, by leveraging a transfer learning framework [66, 67]. Among the five models, the highest performance was achieved by the XGBoost algorithm, with an AUROC of 0.926 (95% CI: 0.793–1.0) and a balanced accuracy of 0.860 (95% CI: 0.636–1.0). In fact, XGBoost was able to correctly identify 31/32 non-MES GBMs and 3/4 MES GBM. This result underscores the strength of the model, as XGBoost successfully classified patients using features derived from the xenograft dataset. Furthermore, the randomized label experiment further supports our findings by showing a drop in model performance when randomizing training label, indicating that the XGBoost model relies on genuine relationships in the data when properly trained. Compared to the study by Le et al [22], who utilized conventional MRI and a clinical patient-to-patient model and an external test set composed of 34 patients (12 MES *versus* 22 non-MES), our binary classification model demonstrated higher performance compared with an AUROC of 0.763 and an overall accuracy of 73.3% for MES subtype identification in a multiclass classification setting. Notably, although their test set was more balanced, our results are still robust despite employing a complex preclinical-to-clinical transfer learning approach.

A key limitation of our study is the small patient cohort, particularly the class imbalance between MES (*n* = 4) and non-MES (*n* = 32) cases, which reflects the natural prevalence of MES in GBM populations. We employed strategies to mitigate potential bias and ensure robustness. Model training was performed exclusively on a xenograft dataset with a balanced distribution (12 non-MES *versus* 9 MES), using stratified 4-fold cross-validation to maintain class balance across folds. The patient dataset was used exclusively as an independent hold-out test set, preserving the integrity of our external validation. We also selected evaluation metrics designed to account for imbalance, reporting balanced accuracy as our primary metric alongside Matthew’s correlation coefficient, while providing the confusion matrix and 95% CIs *via* bootstrap resampling to capture uncertainty. Finally, we performed a randomized label control experiment, which led to a marked reduction in balanced accuracy (0.62 *versus* 0.86). A further general challenge is that individual patient prediction of MES status using presurgical DTI and NODDI remains difficult. Nevertheless, while NODDI acquisition and analysis protocols are not yet widely adopted, emerging acceleration algorithms for multishell dMRI acquisitions and advances in computational analysis could enhance the clinical accessibility of this technique.

Notwithstanding these shortcomings, our findings provide an interesting proof-of-concept of preclinical advanced diffusion MRI imaging biomarkers to clinical settings. Future works should prioritize enhancing the generalizability of our findings by: (1) conducting nested cross-validation on larger, more balanced patient cohorts; (2) incorporating regularization strategies such as L1 or L2 penalties [67] or embedded feature selection methods within the modeling pipeline; and (3) pursuing external validation through multiinstitutional collaborations.

In conclusion, to the best of our knowledge, this is the first study to integrate DTI and NODDI-based radiomics with transcriptomic GBM classification and the first to apply a preclinical-to-clinical transfer learning strategy in this context. The findings not only provide a noninvasive approach for GBM subtype prediction but also establish a methodological blueprint for biomarker translation across species.

## Supplementary information

**Additional file 1:** **Fig. S1.** Transcriptional subgroup affiliation of human GBMs based on an immunohistochemistry (IHC) panel. **Fig. S2.** Kaplan-Meier curves for the GBM patients’ cohort. **Fig. S3.** Transcriptional subgroup affiliation of GSC-derived xenografts based on an immunohistochemistry (IHC) panel. **Fig. S4.** Performance of the 5 prediction models in predicting the molecular subgroup affiliation of GBM patients based on selected aMRI features on patient’s dataset by a Randomized Label Control experiment. **Table S1.** Transcriptional subgroup affiliation of patients affected by WHO Grade 4 gliomas. **Table S2.** Significant radiomic features discriminating MES *versus* non-MES xenografts (B0 mask) after multicollinearity reduction using the Variance Inflation Factor (VIF, threshold > 10) and selected based on a univariate analysis of variance (ANOVA) assessing group differences, retaining only those with *p*-values < 0.05. **Table S3.** Impact of NODDI-derived features on XGBoost model performance for MES prediction. **Table S4.** Performance of the 5 prediction models in predicting the molecular subgroup affiliation of GBM patients based on aMRI features after sanity check. **Table S5.** SHAP values for each of the 9 features.

## Data Availability

All data and materials will be made available upon request.

## Abbreviations

AUROC: Area under the receiver operating characteristic curve
CI: Confidence interval
CL: Classical (glioblastoma subtype)
DTI: Diffusion tensor imaging
EGFR: Epidermal growth factor receptor
FA: Fractional anisotropy
F_ECV_: Fraction of extraneurite volume
F_ICV_: Fraction of intraneurite (intracellular) volume
F_ISO_: Isotropic Gaussian volume fraction
FLAIR: Fluid-attenuated inversion-recovery
FSL: Functional MRI of the Brain—FMRIB (Oxford Centre) Software Library
GBM: Glioblastoma
GLCM: Gray-level co-occurrence matrix
GSC: Glioblastoma sphere-forming cell
IDH: Isocitrate dehydrogenase
IMC1: Informational measure of correlation 1
MD: Mean diffusivity
MES: Mesenchymal (glioblastoma subtype)
MET: MET proto-oncogene
MRI: Magnetic resonance imaging
NODDI: Neurite orientation dispersion and density imaging
NSG: NOD SCID GAMMA
ODI: Orientation Dispersion Index
PN: Proneural (glioblastoma subtype)
ROI: Region of interest
SHAP: Shapley additive explanations
VIF: Variance inflation factor
XGBoost: eXtreme gradient boosting
