# Supplementary material for: Non-invasive identification of mesenchymal glioblastoma using quantitative radiomic features from advanced diffusion MRI: a preclinical-to-clinical transfer learning strategy
Source: Eur Radiol Exp. 2025 Nov 14;9:111. doi: 10.1186/s41747-025-00652-4 (PMC12618778; doi:10.1186/s41747-025-00652-4)
Supplement: Supplementary file 1 — Additional file 1: Fig. S1. Transcriptional subgroup affiliation of human GBMs based on an immunohistochemistry (IHC) panel. Fig. S2. Kaplan-Meier curves for the GBM patients’ cohort. Fig. S3. Transcriptional subgroup affiliation of GSC-derived xenografts based on an immunohistochemistry (IHC) panel. Fig. S4. Performance of the 5 prediction models in predicting the molecular subgroup affiliation of GBM patients based on selected aMRI features on patient’s dataset by a Randomized Label Control experiment. Table S1. Transcriptional subgroup affiliation of patients affected by WHO Grade 4 gliomas. Table S2. Significant radiomic features discriminating MES versus non-MES xenografts (B0 mask) after multicollinearity reduction using the Variance Inflation Factor (VIF, threshold > 10) and selected based on a univariate analysis of variance (ANOVA) assessing group differences, retaining only those with p-values < 0.05. Table S3. Impact of NODDI-derived features on XGBoost model performance for MES prediction. Table S4. Performance of the 5 prediction models in predicting the molecular subgroup affiliation of GBM patients based on aMRI features after sanity check. Table S5. SHAP values for each of the 9 features. [file 41747_2025_652_MOESM1_ESM.pdf]

Non-invasive identification of mesenchymal glioblastoma using quantitative radiomic features from advanced diffusion MRI: a preclinical-to-clinical transfer learning strategy

ELECTRONIC SUPPLEMENTARY MATERIAL

Eur Radiol Exp (2025) Gallotti AL, Pecco N, Pieri V, et al.

Supplementary Figures

Supplementary Fig. S1 - Transcriptional subgroup affiliation of human GBMs based on an immunohistochemistry (IHC) panel

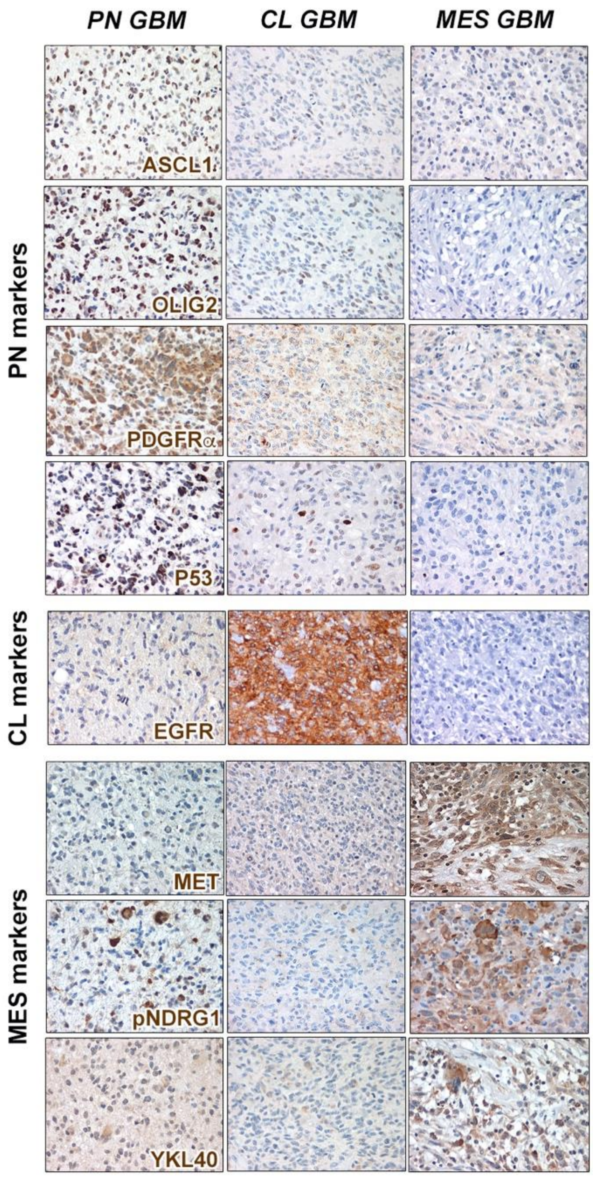

Transcriptional subgroup affiliation of human GBMs was determined through an IHC-based panel that included subgroup-specific gene classifiers (Orzan *et al.,* 2020). The staining for ASCL1, Olig2, PDGFR□, p53, EGFR, MET, pNDRG1 and YKL40 was quantified to release the percentage for each component, thus determining the molecular affiliation. CL: Classical, MES: Mesenchymal, PN: Proneural. Representative PN, CL, and MES GBMs are shown. Magnification: 400x.

Supplementary Fig. S2 - Kaplan-Meier curves for the GBM patients' cohort.

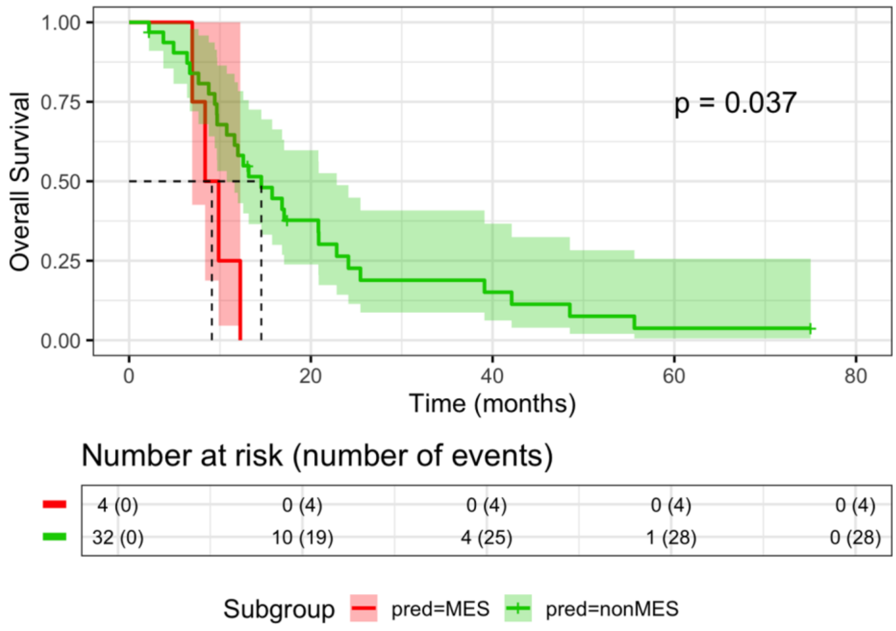

Kaplan–Meier survival analysis of GBM patients stratified according to the different molecular subgroups and grouped as MES *versus* nonMES. Log rank statistics is reported.

Supplementary Fig. S3 - Transcriptional subgroup affiliation of GSC-derived xenografts based on an immunohistochemistry (IHC) panel

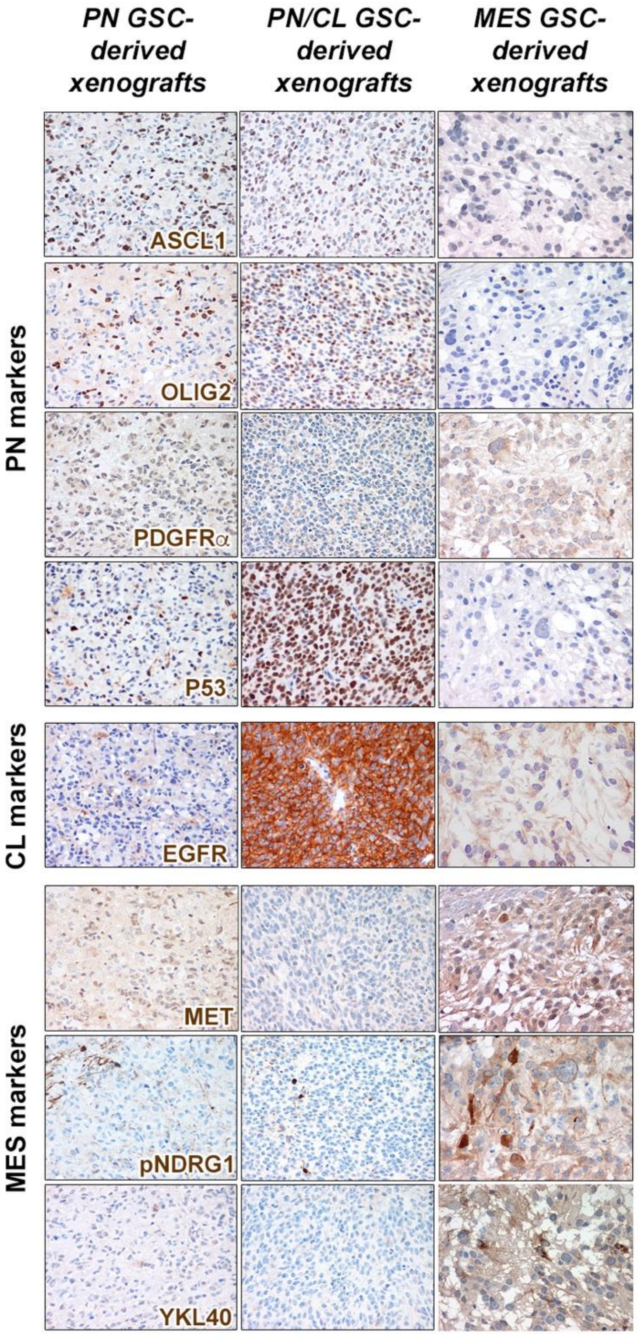

Transcriptional subgroup affiliation of human GSC-derived xenografts was determined through an IHC-based panel that included subgroup-specific gene classifiers (Orzan *et al.,* 2020). The staining for ASCL1, Olig2, PDGFR□, p53, EGFR, MET, pNDRG1 and YKL40 was quantified to release the percentage for each component, thus determining the molecular affiliation. CL: Classical, MES: mesenchymal, PN: proneural. Representative PN, PN/CL, and MES GSC-derived xenografts are shown. Magnification: 400x.

Supplementary Fig. S4 - Performance of the 5 prediction models in predicting the molecular subgroup affiliation of GBM patients based on selected aMRI features on patient's dataset by a Randomized Label Control experiment

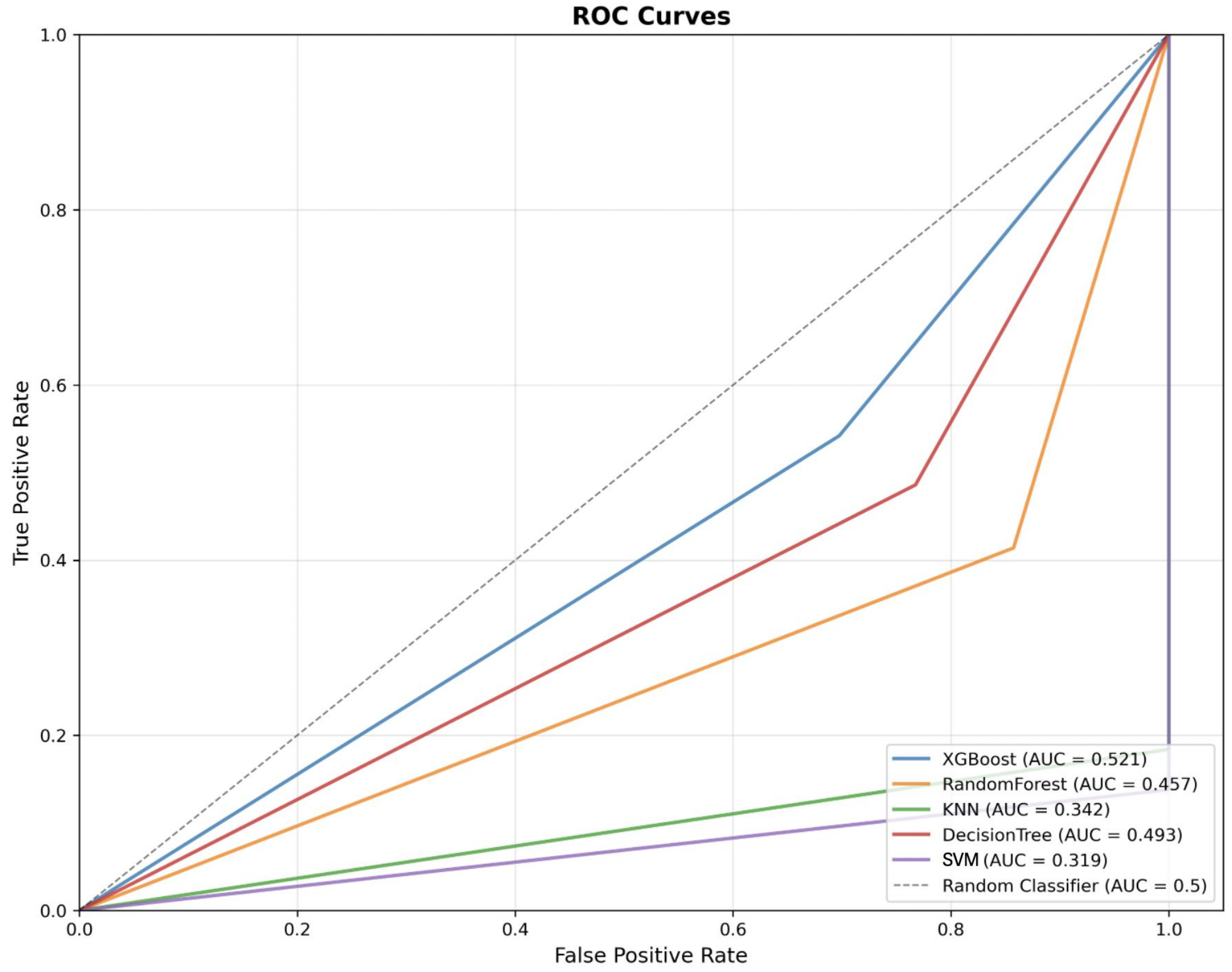

Mean ROC curve of the models. Models were tuned on xenograft values (train dataset) and tested on patients (test dataset). DT: decision tree; SVM: support vector machine; KNN: k-nearest neighbour; RF: random forest; XGBoost: eXtreme Gradient Boosting.

Supplementary Tables

Supplementary Table S1 - Transcriptional subgroup affiliation of patients affected by WHO Grade 4 gliomas

	Sample ID	% PN	% CL	% MES	Affiliation	aMRI
1.	GBM 160315	9.2	1.4	89.4	**MES**	Yes
2.	GBM 160407	75.0	20.0	5.0	**PN**	Yes
3.	GBM 160411	36.6	62.4	1.0	**CL**	Yes
4.	GBM 160503	28.2	69.4	2.4	**CL**	Yes
5.	GBM 160525	63.4	34.2	2.4	**PN**	Yes
6.	GBM 161103	13.8	26.0	60.2	**MES**	Yes
7.	GBM 161116	85.8	7.0	7.2	**PN**	Yes
8.	GBM 161205	53.0	44.6	2.4	**PN/CL**	Yes
9.	GBM 161128	17.8	82.0	0.2	**CL**	Yes
10.	GBM 170413	3.8	96.2	0.0	**CL**	Yes
11.	GBM 170421	9.0	91.0	0.0	**CL**	Yes
12.	GBM 170426	59.2	38.0	2.8	**PN**	Yes
13.	GBM 171025	11.8	88.2	0.0	**CL**	Yes
14.	GBM 171027	24.4	74.6	1.0	**CL**	Yes
15.	GBM 171031	14.0	85.6	0.4	**CL**	Yes
16.	GBM 171211	33.6	65.4	1.0	**CL**	Yes
17.	GBM 180111	21.8	77.8	0.4	**CL**	Yes
18.	GBM 180510	55.4	39.4	5.2	**PN/CL**	Yes
19.	GBM 191119	60.2	37.2	2.6	**PN**	Yes
20.	GBM 190920	33.6	65.4	1.0	**CL**	Yes
21.	GBM 180420	20.2	5.2	74.6	**MES**	Yes
22.	GBM 180522	70.2	27.8	2.0	**PN**	Yes
23.	GBM 180515	54.8	42.0	3.2	**PN/CL**	Yes
24.	GBM 180507	46.0	26.2	27.8	**MIXED**	Yes
25.	GBM 171214	51.0	43.2	5.8	**PN/CL**	Yes
26.	GBM 180611	77.2	19.4	3.4	**PN**	Yes
27.	GBM 190724	54.6	40.6	4.8	**PN/CL**	Yes
28.	GBM 181224	80.4	17.6	2.0	**PN**	Yes
29.	GBM 190222	67.0	27.8	5.2	**PN**	Yes
30.	GBM 190207	34.8	64.0	1.2	**CL**	Yes
31.	GBM 181012	43.4	30.2	26.4	**MIXED**	Yes
32.	GBM 190515	57.4	38.2	4.4	**PN**	Yes
33.	GBM 201113	67.0	27.6	5.4	**PN**	Yes
34.	GBM 190628	35.6	63.2	1.2	**CL**	Yes
35.	GBM 181123	56.0	41.6	2.4	**PN/CL**	Yes
36.	GBM 200310	11.4	20.4	68.2	**MES**	Yes

The percentage of cells expressing PN, CL or MES markers is reported for each sample, as well as the resulting affiliation according to protocol established by Orzan et al., 2020. GBM: glioblastoma; PN: proneural; CL: classical; MES: mesenchymal; MIXED: balanced mix of the three subgroups.

Supplementary Table S2. Significant radiomic features discriminating MES *versus* non-MES xenografts (B0 mask) after multicollinearity reduction using the Variance Inflation Factor (VIF, threshold >10) and selected based on a univariate analysis of variance (ANOVA) assessing group differences, retaining only those with p-values <0.05.

	Radiomic feature	p-Value
1.	'FA_B0_original_glcm_Correlation'	0.0131
2.	'FA_B0_original_glcm_Imc1'	0.0012
3.	'fecv_B0_original_firstorder_10Percentile'	0.0395
4.	'fecv_B0_original_firstorder_90Percentile'	0.0019
5.	'odi_B0_original_firstorder_Entropy'	0.0046
6.	'odi_B0_original_firstorder_Kurtosis'	0.0104
7.	'odi_B0_original_glcm_ClusterProminence'	0.0077
8.	'odi_B0_original_glcm_Imc1'	0.0002
9.	'odi_B0_original_glrlm_LongRunLowGrayLevelEmphasis'	0.0195

The first component of the feature indicates the diffusion map in which it was significant (FA, MD, fiso, fecv, ficv, odi), the second part which sequence mask was applied to the map (B0), the last part the actual radiomic feature. FA: fractional anisotropy (DTI), fecv: fraction of extraneurite volume (NODDI), ficv: fraction of intraneurite volume (NODDI), fiso: fraction of isotropic diffusion (NODDI), MD: mean diffusivity (DTI), odi: orientation dispersion index (NODDI).

Supplementary Table S3 - Impact of NODDI-derived features on XGBoost model performance for MES prediction.

	XGBoost	
	**DTI**	**DTI + NODDI**
**Balanced Accuracy**	0.860 (0.781–0.933)	0.860 (0.636–1.000)
**Precision Macro**	0.664 (0.571–0.792)	**0.871 (0.636–1.000)**
**Recall Macro**	0.860 (0.781–0.933)	0.860 (0.636–1.000)
**F1 Macro**	0.658 (0.516–0.821)	**0.851 (0.636–1.000)**
**Matthews Corrcoef**	0.480 (0.204–0.695)	**0.720 (0.273–1.000)**
**Roc Auc**	0.856 (0.707–0.976)	**0.926 (0.793–1.000)**

Performance metrics (mean and 95% confidence intervals) of the XGBoost classifier trained on the xenograft dataset and tested on the independent patient cohort, comparing a model using only DTI-derived features versus a model including both DTI and NODDI features.

Supplementary Table S4 - Performance of the 5 prediction models in predicting the molecular subgroup affiliation of GBM patients based on aMRI features after sanity check.

	XGBoost Mean (95% CI)	RandomForest Mean (95% CI)	KNN Mean (95% CI)	DecisionTree Mean (95% CI)	SVM Mean (95% CI)
**Balanced Accuracy**	0.623 (0.500–0.871)	0.484 (0.448–0.500)	0.389 (0.312–0.453)	0.500 (0.500–0.500)	0.251 (0.167–0.339)
**Precision Macro**	0.788 (0.417–0.986)	0.439 (0.389–0.472)	0.426 (0.365–0.468)	0.059 (0.028–0.111)	0.394 (0.300–0.455)
**Recall Macro**	0.623 (0.500–0.871)	0.484 (0.448–0.500)	0.389 (0.312–0.453)	0.500 (0.500–0.500)	0.251 (0.167–0.339)
**F1 Macro**	0.648 (0.455–0.918)	0.460 (0.420–0.486)	0.406 (0.345–0.454)	0.104 (0.053–0.182)	0.304 (0.217–0.379)
**Matthews Corrcoef**	0.378 (0.000–0.848)	-0.048 (-0.130–0.000)	-0.176 (-0.297–-0.091)	0.000 (0.000–0.000)	-0.319 (-0.491–-0.187)
**Roc Auc**	0.521 (0.152–0.897)	0.457 (0.089–0.848)	0.342 (0.122–0.574)	0.493 (0.221–0.774)	0.319 (0.091–0.554)

Models were tuned on xenograft values (train dataset) and tested on patients (test dataset). LR: linear regression; SVM: support vector machine; KNN: k-nearest neighbour; RF: random forest; XGBoost: eXtreme Gradient Boosting.

Supplementary Table S5 – SHAP values for each of the 9 features.

Feature	SHAP Importance
**fecv_B0_original_firstorder_90Percentile**	0.466
**FA_B0_original_glcm_Imc1**	0.435
**odi_B0_original_glcm_Imc1**	0.428
**odi_B0_original_firstorder_Entropy**	0.100
**fecv_B0_original_firstorder_10Percentile**	0.089
**odi_B0_original_glcm_ClusterProminence**	0.073
**FA_B0_original_glcm_Correlation**	0.060
**odi_B0_original_glrlm_LongRunLowGrayLevelEmphasis**	0.054
**odi_B0_original_firstorder_Kurtosis**	0.049

SHAP values were extracted when predicting patients hold out test set form the eXtreme Gradient Boosting best performing model.

Supplementary Schematic 1

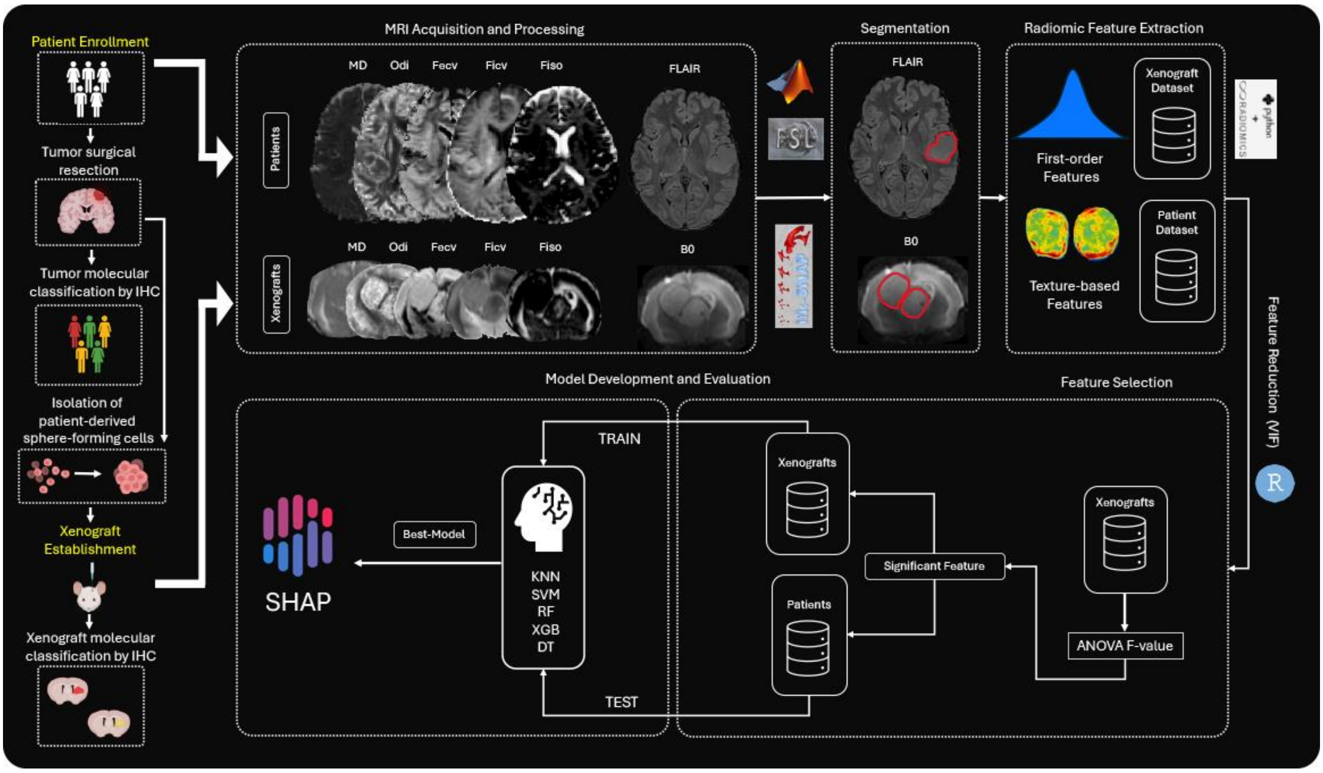

A schematic summarizing the experimental pipeline of the study.

Supplementary Methods

Glioblastoma sphere-forming cell (GSC) propagation in vitro

GSC lines were cultured using Dulbecco's Modified Eagle Medium−DMEM-F12 (Invitrogen) supplemented with 2% Glutamax (Invitrogen), penicillin/streptomycin (100 units/ml penicillin, 100 µg/ml streptomycin - Invitrogen), glucose 0.6% (Sigma-Aldrich), HEPES 5 mM (Invitrogen), $NaHCO_3$ 0·11% (Invitrogen), Hormone Mixture (B27 analogue, homemade), Heparin 0.2% (Sigma-Aldrich), EGF 20 ng/ml (Peprotech), FGF2 10 ng/ml (Peprotech).

The hormone mixture contained Dulbecco's Modified Eagle Medium−DMEM-F12, glucose 0.6% (Sigma-Aldrich), HEPES 5 mM (Invitrogen), NaHCO3 0.11% (Invitrogen), 77.2 mg/ml putrescine (Sigma-Aldrich), 0.5 mg/ml insulin (Sigma-Aldrich), 100 mg/ml apo-transferrin (Sigma-Aldrich), sodium selenite 3 mM (Sigma-Aldrich), progesterone 2 mM (Sigma-Aldrich).

Neurospheres were harvested and centrifuged, and the pellet mechanically dissociated to obtain a single cell suspension that was again plated in culture medium to allow for GSC line propagation.

Immunohistochemistry-based analysis

Immunohistochemical staining was performed on 2 µm sections that were cut from paraffin blocks, deparaffinized and rehydrated. The endogenous peroxidase activity was blocked with 0.3% H_2O_2 in methanol for 20 minutes. Antigen retrieval was performed using a 0.05% protease type XIV (Sigma-Aldrich) solution at 37°C for 20 minutes or a microwave-oven or a thermostatic bath in either 1·0 mM EDTA buffer (pH 8.0) or 1mM Citrate buffer (pH 6.0). Then slides were washed in TBS (pH 7.4) and incubated for an hour or overnight in the specific primary antibody diluted in TBS 1% Bovine Serum Albumin (BSA) or in Antibody Diluent with Background Reducing Components (DAKO). Sections were washed in TBS and signal revealed using Envision+System-HRP Labelled Polymer Anti-mouse or Anti-Rabbit (DAKO) or Novolink™ Polymer Detection System (Novocastra™) or Rabbit specific HRP/DAB immunohistochemistry detection Kit-Micro-polymer (Abcam), followed by Diaminobenzidine (DAB) as chromogen and Hematoxylin as counterstain.

Images were acquired with a Nikon DS-Ri2 camera (4908x3264 full-pixel) mounted on a Nikon Eclipse 50i microscope equipped with Nikon Plan lenses (x10/0.25; x20/0.40; x40/0.65; x100/1.25) using NIS-Elements 4.3 imaging software (Nikon Corporation).

The primary antibodies used for the staining were:
anti-EGFR (clone E30 #M7239, Dako, Glostrup, Denmark, USA, RRID:AB_2721108, 1:20), anti-p53 (clone DO-7 #MS-186-P, Thermo Scientific, Waltham, MA, USA, RRID:AB_62273, 1:2), anti-PDGFRA (#PA5-16742, Thermo Scientific, Waltham, MA, USA,

RRID:AB_10980345, 1:50), anti-Olig2 (#AB9610 Chemicon, Darmstadt, Germany, RRID:AB_570666, 1:600), anti-ASCL1 (clone 24B72D11.1 #556604, BD Biosciences, Franklin Lakes, NJ, USA RRID:AB_396479, 1:50), anti-pNDRG1 (Thr346, clone D98G11 #5482, Cell Signalling, Beverly, MA, USA, RRID:AB_10693451, 1:100), anti-YKL40 (#4815, Quidel, San Diego, CA, USA, RRID:AB_452475, 1:100), anti-MET (C-12 #sc-10, Santa Cruz Biotechnology, Dallas, Texan, USA, RRID:AB_631940, 1:50).

Expression of subgroup-specific gene classifiers was scored to calculate the PN, CL, and MES percent component in each sample. A neat affiliation was defined if the prevalent component exceeded the second-prevalent by > 20%; otherwise, the affiliation was defined as a mixture of the two most highly represented components, or as a mixture of the three subgroups if they were equally represented.

Patient MRI acquisition

GBM patient datasets were acquired on a 3T Ingenia CX scanner (Philips Healthcare, Best, The Netherlands), using a 32-channel head coil. Conventional MRI protocol included an axial 3D fluid attenuated inversion recovery (3D-FLAIR) (TR/TE/TI 9,000/290/2,500 ms; flip angle, 40; 204 slices; thickness, 0·7/−0·5 mm gap; matrix, 204×197; SENSE reduction factor R=2; acquisition time 7min 30s). The NODDI protocol consisted in a two-shell acquisition based on axial single-shot spin-echo echo planar imaging with an anterior-posterior phase-encoding direction that included:

• HARDI: 60 diffusion-weighted volumes (diffusion gradients along 60 noncollinear directions; b-value 3.000 s/mm2).

• DTI: 35 diffusion-weighted volumes (diffusion gradients along 35 noncollinear directions; b-value 711 s/mm2).

• 11 “B0” volumes without diffusion-weighting (b-value, 0 s/mm2).

A “reverse B0” without diffusion-weighting was acquired (b-value 0 s/mm2), which shared with the NODDI sequence all the geometrical features but the phase-encoding direction that was posterior-anterior to allow for susceptibility artifacts correction[39].

Xenograft MRI acquisition

Xenograft MRI acquisition was carried out on a 7 Tesla preclinical scanner (Bruker, BioSpec 30/70 USR, Paravision 5.1, Germany) at OSR Experimental Imaging Centre (EIC), equipped with 450/675 mT/m gradients (slew-rate: 3400-4500T/m/s; rise-time: 140ms). A phased-array mouse-head coil with 4 internal preamplifiers was used as receiver, coupled with a 72 mm linear-volume coil as transmitter. Mice were anaesthetized with Sevoflurane® (5% for induction, 2% for maintenance) in a 95-98% O2 mixture under temperature control. Xenograft-bearing mice underwent the same advanced imaging protocol used for patients.

Radiomic features

As reported in the main manuscript, radiomic features were extracted from each diffusion map by using the 3D-FLAIR and post-Gd T1 masks in patients and the B0 image in xenografts.

The strength of the image texture features extracted from MRI images lies in their ability to capture subtle variations in the spatial distribution of pixel intensities that are not easily discernible by the naked eye. These variations can be indicative of underlying tissue characteristics, such as heterogeneity, necrosis, or vascularity, which may not be captured by traditional radiological measurements, such as tumor size or volume.

Histogram features are 18 and describe the distribution of pixel intensities within the ROI, which can provide information about the overall brightness or contrast of the image. This can be useful in identifying regions of necrosis, which typically have low pixel intensity values, or regions of high vascularity, which can have high pixel intensity values.

Twenty-two grey-level co-occurrence matrix (GLCM) features describe the probabilities of co-occurrence of pixel pairs with given grey levels, while 16 grey-level run-length matrix (GLRLM) features represent runs of pixels having the same grey-level value. These can be useful in identifying regions of heterogeneity within the ROI, as regions with high heterogeneity would have a greater variety of co-occurring pixel pairs or may have more frequent changes in grey-level values.

Sixteen size zone matrix features characterize pixel zones in the images, which can provide information about the size and shape of regions of interest. This can be useful in identifying regions of necrosis or tumor infiltration, which typically have irregular shapes and sizes.

Fourteen grey-level dependence matrix (GLDM) features quantify the level of dependence in an image, which can be useful in identifying patterns of vascularity or tissue heterogeneity. Regions with high vascularity, for example, may have a greater degree of dependence among pixel values.

Finally, five neighbouring-grey-tone difference matrix (NGTDM) features explain the difference between a grey value and its neighbours, which can be useful in identifying regions of tissue heterogeneity or necrosis. Regions with high heterogeneity, for example, may have greater differences in grey values among neighbouring pixels.

Shape-based features were not calculated due to different dimensions of ROIs and manual lesions segmentation.

Model Development

The predictive models were trained on the xenograft dataset and then tested on the patient hold-out test set. Xenograft features were normalized (Z-score) and used as the training data, given that in xenografts tissue heterogeneity is more consistent, and the impact of

confounding clinical variables is minimized. This enables more robust training of the model on high-quality, well-characterized radiomic features. On the other hand, testing the model on the patient dataset served as a critical validation step, ensuring that the predictive power of the radiomic features can be translated to human subjects, who exhibit greater clinical variability.

Supplementary Results

Molecular, demographic, and clinical information of the patients' cohort

According to the inclusion and exclusion criteria described in the Methods' section, we enrolled 36 isocitrate dehydrogenase 1/2 wild-type GBM patients (Schematic 1). This cohort consisted of 13 females (36·1%) and 23 males (63·9%), with a mean age of 62·6 ± 10·4 years (median 63·5, range 41-80 years). Mean preoperative Karnofsky Performance Scale (KPS) was 90·8 ± 9·7 (median 90, range 70-100). Sixteen patients (44·4%) were diagnosed due to seizure onset, while the remaining 20 following non-specific symptoms or development of stable neurological deficits. In total, 28 (77·8%) patients had preoperative neurological deficits, either mild (*e.g.,* anomia and/or paraphasia, limited quadrantanopia) or severe (*e.g.,* complete aphasia or severe hemiparesis). Fourteen lesions (38·9%) were right-sided, 21 (58·3%) left-sided, and 1 (2·8%) diffused on both sides. Two patients underwent stereotactic biopsy only. Three patients had previous open surgeries with diagnosis of GBM in 2 cases and anaplastic astrocytoma in the remaining one; two of them were treated with chemo-radiotherapy, before enrollment in our study. Thirty-four patients underwent open surgery, with 27 receiving gross-total resections (79·4%), 6 subtotal resections (<10% residue, 17·6%), and 1 partial resections (>10% residue, 2·9%).

Interpretation of selected radiomic features

Violin plots in Figure 2 shows standardized (z-scored) xenografts radiomic features distributions between mesenchymal and non-mesenchymal. Higher values of the first-order FECV percentiles (10th and 90th) indicates a significantly hindered diffusion component. Increased ODI entropy corresponds to greater disorder in tissue orientation, while elevated ODI kurtosis reflects more extreme orientation values. Regarding GLCM-derived texture features, higher Imc1 values likely related to textural complexity in both diffusion anisotropy and neurite dispersion patterns, with greater microstructural heterogeneity. Similarly, Cluster Prominence captures regional heterogeneity through intensity clustering patterns - higher values indicate more distinct, well-separated tissue regions with different imaging characteristics. The difference between Imc1 and cluster prominence is that Imc1 quantifies local pixel-to-pixel relationships and fine-scale textural complexity, while cluster prominence measures the distinctiveness of broader regional intensity patterns. This explains why

mesenchymal tissues can simultaneously exhibit high local complexity (elevated Imc1) within relatively uniform regional organization (lower cluster prominence), while non-mesenchymal tissues show simpler local relationships (lower Imc1) but more pronounced regional compartmentalization (higher cluster prominence). Among higher-order features, low values of long run low grey level emphasis (GLRLM) highlights lacking long, continuous stretches of uniformly organized fibers, indicating interrupted fiber organization.
